# 超高效液相色谱-串联质谱法测定尿液中118种农药残留

**DOI:** 10.3724/SP.J.1123.2023.04001

**Published:** 2024-01-08

**Authors:** Wei SONG, Kaiyong LIU, Lijun CHEN, Yu WANG, Yachao NI, Yarong HU, Xueying JIA, Fang HAN, Yuxin LIU, Dianbing ZHOU

**Affiliations:** 1.人口健康与优生安徽省重点实验室(安徽医科大学), 安徽 合肥 230032; 1. Anhui Provincial Key Laboratory of Population Health & Aristogenics (Anhui Medical University), Hefei 230032, China; 2.合肥海关技术中心, 安徽 合肥 230022; 2. Technical Center for Hefei Customs, Hefei 230022, China; 3.安徽医科大学公共卫生学院, 安徽 合肥 230032; 3. School of Public Health, Anhui Medical University, Hefei 230032, China

**Keywords:** QuEChERS, 超高效液相色谱-串联质谱, 农药, 尿液, 多残留, QuEChERS, ultra-high performance liquid chromatography-tandem mass spectrometry (UHPLC-MS/MS), pesticides, urine, multi-residues

## Abstract

为有效监测人尿液中的农药多残留水平,为健康风险评估提供重要的技术手段,实验利用QuEChERS前处理方法结合超高效液相色谱-三重四极杆质谱技术建立了尿液中118种农药的快速筛查及测定方法。通过对前处理过程、液相色谱分离和质谱条件的系统优化,实现了在2 h内对样品中118种目标分析物的提取及分析测定。具体方法如下:尿液样品中农药目标分析物采用乙腈提取,无水MgSO_4_加NaCl作除水盐析剂,再以C_18_、PSA、无水MgSO_4_为净化吸附剂,经QuEChERS法净化,氮吹复溶后,以0.01%甲酸水溶液(含2 mmol/L甲酸铵)及0.01%甲酸甲醇溶液(含2 mmol/L甲酸铵)作为流动相,ZORBAX Eclipse Plus C_18_柱(100 mm×2.1 mm, 1.8 μm)作为分析色谱柱,梯度洗脱分离,超高效液相色谱-三重四极杆质谱正负离子切换动态多反应监测(DMRM)模式检测,外标法定量。结果表明,该方法可以对尿液中的118种农药同时进行快速测定,检出限均可达到0.10 μg/L,定量限均可达到0.50 μg/L,基质效应均小于20%。在0.50、1.00、5.00 μg/L 3个添加水平下,尿样中118种农药残留的平均回收率为70.2%~104%,相对标准偏差(RSD)为2.8%~9.3%。采用本方法对10份尿液样品进行检测,共检出噻虫嗪、噻虫胺、啶虫脒、呋虫胺、异丙隆、烯酰吗啉6种农药,含量均≤3.65 μg/L。检出的农药噻虫嗪、噻虫胺与当前该类农药在农产品中的使用情况关联性较高。该方法高效、灵敏、准确,可用于人尿液样品中118种农药残留的快速筛查测定。

## Abstract (en)

Pesticide residues in food and their hazardous effects have attracted much attention given the increased and widespread use of pesticides. The long-term consumption of food containing pesticide residues is an important pathway for the gradual accumulation of pesticides in the human body. Urine is often monitored as a biological sample for low-dose exposure to pesticides, and urine collection is a relatively convenient sampling technique in general population research. In order to effectively monitor residual levels of multiple pesticides in human urine and provide an important technological approach for health risk assessment, a rapid screening and confirmatory detection method for 118 pesticides in urine was established using QuEChERS method as a pretreatment combined with ultra-high performance liquid chromatography-triple quadrupole mass spectrometry (UHPLC-MS/MS). The 118 pesticides analyzed included organophosphorus, carbamate, neonicotinoid, and strobilurin fungicides and other widely used pesticides. Following systematic optimization of the pretreatment process, LC separation conditions, and MS/MS parameters, 118 pesticides were extracted from urine samples and analyzed within 2 h. In brief, the target analytes in 5 mL urine samples were extracted with 10 mL of acetonitrile and added with 5 g of anhydrous MgSO_4_ and 1 g of NaCl as water-removal and salting-out agents, respectively. After centrifugation, 6 mL of the supernatant was cleaned using the QuEChERS method with 300 mg of C_18_, 300 mg of primary secondary amine (PSA) and 900 mg of anhydrous MgSO_4_ as the purification adsorbent. After nitrogen blowing and solubilization, the 118 target analytes were separated on a ZORBAX Eclipse Plus C_18_ analytical chromatographic column (100 mm×2.1 mm, 1.8 μm) with gradient elution using (A) 0.01% formic acid aqueous solution (containing 2 mmol/L ammonium formate) and (B) 0.01% formic acid methanol solution (containing 2 mmol/L ammonium formate) as mobile phases. The gradient elution program was as follows: 0-0.5 min, 5%B; 0.5-1.5 min, 5%B-20%B; 1.5-2.5 min, 20%B-50%B; 2.5-8.0 min, 50%B-80%B; 8.0-9.0 min, 80%B-98%B; 9.0-11.0 min, 98%B; 11.0-11.5 min, 98%B-5%B; 11.5-15.0 min, 5%B. The analytes were then determined by UHPLC-MS/MS with positive/negative ion switching in dynamic multiple-reaction monitoring mode and quantified using the external standard method. The results indicated that the proposed method can determine 118 pesticides in urine simultaneously and rapidly. The limits of detection and limits of quantification were 0.10 and 0.50 μg/L, respectively, and the matrix effects were less than 20%for all targeted compounds. The recoveries of the 118 pesticides in urine were between 70.2% and 104% at three spiked levels of 0.50, 1.00, and 5.00 μg/L, and the relative standard deviations ranged from 2.8% to 9.3%. The method was applied to 10 actual urine samples, and the results revealed the presence of six pesticides, including thiamethoxam, clothianidin, acetamiprid, dinotefuran, isoproturon, and dimethomorph, with contents ranging from <LOQ to 3.65 μg/L. Thiamethoxam and clothianidin were detected most often in the samples, and their concentrations were highly correlated with the current use of these pesticides in edible agricultural products. The proposed method has the characteristics of high efficiency, sensitivity, and accuracy and is suitable for the rapid screening and determination of the 118 pesticide residues in human urine samples.

农药在农业生产中使用量较高^[1,2]^,长期食用含有农药残留的食品是农药在人体内逐渐蓄积的一大重要途径^[3]^。日常生活中摄入带有微量农药残留的食物虽然不能直接导致明显伤害,但通过在人体内的代谢、吸收和富集,会对健康造成潜在的威胁,如癌变、神经系统失调和畸变等^[4⇓-6]^。

尿液常作为农药低剂量暴露的被监测生物样本,在一般人群研究中,现场尿样收集相对方便,目前国内外已有很多学者针对人体尿液中农药残留开展了相关研究。Luiz等^[7]^利用枪头式分散固相微萃取技术,结合气相色谱-质谱法研究建立了人群尿液中杀线威、残杀威、克百威、3-羟基克百威、西维因、甲硫威、特丁硫磷、甲基对硫磷、马拉硫磷、毒死蜱和硫丹等11种农药的测定方法,检出限为0.76~1.52 μg/L,定量限为2.5~5.0 μg/L。Fierová等^[8]^将QuEChERS与固相萃取技术相结合,采用液相色谱-串联质谱建立了人体尿液中磷酸二乙酯等15种农药代谢物的定性定量检测方法,并对20份南非儿童尿样进行了监测。Iqbal等^[9]^使用改良的QuEChERS法提取,气相色谱-质谱测定,建立了人群血液和尿液中9种农药的测定方法,农药种类涉及有机磷、氨基甲酸酯和拟除虫菊酯等多类农药,定量限均达到0.01 mg/L。金玉娥等^[10]^采用酸水解,液液萃取,液相色谱C_18_柱分离,大气压化学电离源质谱检测,建立了人尿中3种拟除虫菊酯类农药代谢物3-苯氧基苯甲酸(3-phenoxybenzoic acid)和顺式-、反式-3-(2,2-二氯乙烯基)-2,2-二甲基环丙烷-1-羧酸的定量方法,3种代谢物的检出限为0.20 μg/L。

有机磷类、氨基甲酸酯类、拟除虫菊酯类、新烟碱类农药及活性代谢物的大量使用已造成不同人群多种途径的普遍暴露^[11⇓⇓⇓-15]^。以每年市场监督管理局发布的食用农产品抽检信息统计来看,蔬菜、水果、茶叶等食品中农药残留超标时有报道,涉及多类农药。目前针对人体尿样中农药及代谢物残留的检测方法多集中于针对几种或者几类农药残留的测定,而针对人尿液中农药残留高通量检测方法的研究还较少。

本实验以人尿液为研究对象,参考当前食品农药使用情况相关文献报道^[16⇓⇓⇓-20]^、市场监督管理局食用农产品抽检监测信息及实验室农药标准品储备情况,从有机磷类、氨基甲酸酯类、新烟碱类、甲氧基丙烯酸酯类等多个类别农药中选取118种常见农药,以QuEChERS前处理技术为净化手段,采用超高效液相色谱-串联质谱法,建立了尿液中118种农药的快速筛查方法。本法具有快速、灵敏、准确的特点,为人尿液中农药的高通量快速测定提供了可靠的监测技术,以期为后续健康风险评估提供重要技术手段及基础数据。

## 1 实验部分

### 1.1 仪器、试剂与材料

1290-6460超高效液相色谱-三重四极杆质谱仪(美国Agilent公司); Vortex-Genie 2涡旋混匀器(美国Scientific Industries公司);乙腈、甲醇(色谱纯,美国TEDIA公司);甲酸(LC-MS级,美国ACS公司);甲酸铵、盐酸、无水MgSO_4_、NaCl(分析纯,上海国药集团);C_18_、*N*-丙基乙二胺(PSA)(美国Agilent公司);实验用水均为超纯水(电阻率为18.2 MΩ·cm); 118种农药标准品:纯度≥98%(天津阿尔塔科技有限公司)。尿液样品来自于安徽医科大学。本研究方案通过安徽医科大学伦理委员会论证审查(批准号:20189999)。

### 1.2 样品前处理

移取试样5 mL于50 mL塑料离心管中,加入1 mol/L盐酸调节pH至5.0。再加入10 mL乙腈,加盖涡旋振荡2 min后加入5 g无水MgSO_4_、 1 g NaCl,再加盖涡旋振荡1 min,以8000 r/min离心5 min,将6 mL上层清液转移至另一50 mL离心管中,加入300 mg C_18_、300 mg PSA和900 mg无水MgSO_4_,涡旋1 min后以8000 r/min离心5 min,收集3 mL上清液于玻璃离心管中,在40 ℃水浴中用氮气吹干;随后用300 μL初始流动相复溶,过0.22 μm有机相滤膜,供UHPLC-MS/MS分析。

### 1.3 UHPLC-MS/MS条件

#### 1.3.1 色谱条件

色谱柱为ZORBAX Eclipse Plus C_18_柱(100 mm×2.1 mm, 1.8 μm);流动相:A相为0.01%甲酸水溶液(含2 mmol/L甲酸铵), B相为0.01%甲酸甲醇溶液(含2 mmol/L甲酸铵)。梯度洗脱程序:0~0.5 min, 5%B; 0.5~1.5 min, 5%B~20%B; 1.5~2.5 min, 20%B~50%B; 2.5~8.0 min, 50%B~80%B; 8.0~9.0 min, 80%B~98%B; 9.0~11.0 min, 98%B; 11.0~11.5 min, 98%B~5%B; 11.5~15.0 min, 5%B。流速:0.3 mL/min;柱温:35 ℃;进样量10 μL。

#### 1.3.2 质谱条件

离子源:电喷雾电离源(ESI),正负离子切换模式;鞘气温度:320 ℃;鞘气流速:11 L/min;雾化气压力:276 kPa;干燥气温度:350 ℃;干燥气流速:10 L/min;毛细管电压:4000 V。动态多反应监测(DMRM)模式:其他参数见表1。

**表1:** 118种农药的母离子、子离子、碎裂电压、碰撞能量及保留时间

Compound	Parent ion (m/z)	Product ions (m/z)	Fragmentor/V	Collision energies/eV	t_R_/min
Fenpyrazamine (胺苯吡菌酮)	332.0	230.0^*^, 216.0	130	20, 25	7.7
Pyraclostrobin (百克敏)	388.0	163.0^*^, 194.0	120	10, 20	9.1
Fenthion (倍硫磷)	279.0	247.0^*^, 169.0	100	10, 15	7.2
Fenthion sulfoxide (倍硫磷亚砜)	295.0	280.0^*^, 109.0	115	15, 25	5.4
Tribenuron-methyl (苯磺隆)	396.1	155.0^*^, 181.0	75	10, 22	6.1
Metrafenone (苯菌酮)	409.0	209.0^*^, 227.0	100	15, 20	9.3
Difenoconazole (苯醚甲环唑)	406.0	337.0^*^, 251.0	110	15, 25	9.4
Mefenacet (苯噻酰草胺)	299.0	148.1^*^, 120.1	92	9, 25	7.7
Benalaxyl (苯霜灵)	326.1	208.1^*^, 148.1	88	9, 17	8.9
Zoxamide (苯酰菌胺)	336.1	187.0^*^, 159.0	110	17, 40	9.0
Fenamiphos (苯线磷)	304.1	216.9^*^, 202.0	158	21, 38	8.4
Imidacloprid (吡虫啉)	256.0	209.1^*^, 175.1	88	13, 17	3.8
Diflufenican (吡氟酰草胺)	395.0	266.0^*^, 246.0	120	25, 30	9.5
Penthiopyrad (吡噻菌胺)	360.1	275.9^*^, 255.9	110	12, 20	8.7
Metazachlor (吡唑草胺)	278.1	134.1^*^, 210.1	70	22, 4	6.2
Isopyrazam (吡唑萘菌胺)	360.0	320.0^*^, 244.1	110	20, 20	9.4
Prosulfocarb (苄草丹)	252.1	128.0^*^, 91.1	92	6, 17	9.6
Bensulfuron-methyl (苄嘧磺隆)	411.1	149.1^*^, 182.1	112	13, 13	6.8
Pretilachlor (丙草胺)	312.1	252.0^*^, 176.1	112	29, 10	9.6
Propyrisulfuron (丙嗪嘧磺隆)	456.1	261.0^*^, 196.0	110	10, 10	7.7
Propoxur (残杀威)	210.1	168.0^*^, 111.0	64	5, 9	5.2
Tebufenozide (虫酰肼)	353.1	133.0^*^, 297.0	80	13, 2	8.4
Pyridaphenthion (哒嗪硫磷)	341.0	205.0^*^, 189.0	118	22, 18	7.7
Isoprothiolane (稻瘟灵)	291.0	231.0^*^, 188.9	84	5, 21	7.5
Edifenphos (敌瘟磷)	311.1	283.0^*^, 111.0	110	10, 20	8.7
Tebuthiuron (丁噻隆)	229.1	172.1^*^, 116.0	110	17, 25	5.4
Acetamiprid (啶虫脒)	223.0	126.0^*^, 56.0	108	21, 13	4.0
Picoxystrobin (啶氧菌酯)	368.1	205.0^*^, 145.0	74	5, 17	8.4
Chlorfenvinphos (毒虫畏)	359.1	127.0^*^, 155.0	120	15, 10	8.9
Carbendazim (多菌灵)	192.0	160.0^*^, 132.0	118	17, 33	2.7
Metamifop (噁唑酰草胺)	441.1	288.0^*^, 123.0	121	18, 30	9.7
Diazinon (二嗪磷)	305.1	169.0^*^, 153.0	140	20, 20	8.9
Fensulfothion (丰索磷)	309.0	253.0^*^, 157.0	132	14, 29	6.2
Dinotefuran (呋虫胺)	203.2	129.2^*^, 87.1	80	5, 10	0.7
Furathiocarb (呋线威)	383.1	252.0^*^, 195.0	102	9, 13	9.8
Flucetosulfuron (氟吡磺隆)	488.1	156.0^*^, 273.0	116	18, 26	7.0
Fluopicolide (氟吡菌胺)	382.9	172.9^*^, 144.9	110	32, 42	7.5
Fluopyram (氟吡菌酰胺)	397.1	173.1^*^, 208.0	107	29, 17	7.8
Fipronil (氟虫腈)	434.9	250.0^*^, 330.0	-120	-30, -15	8.4
Fipronil sulfone (氟虫腈砜)	450.9	282.0^*^, 415.0	-135	-30, -15	8.8
Flusilazole (氟硅唑)	316.1	247.0^*^, 165.0	126	13, 29	8.4
Triflumizole (氟菌唑)	346.1	278.0^*^, 73.0	80	5, 10	9.6
Flufenacet (氟噻草胺)	364.1	152.0^*^, 194.0	80	10, 5	8.0
Flutolanil (氟酰胺)	324.1	282.1^*^, 262.1	152	10, 14	7.5
Fluxapyroxad (氟唑菌酰胺)	382.1	362.1^*^, 234.1	110	12, 24	7.5
Silthiofam (硅噻菌胺)	268.1	139.1^*^, 73.0	135	16, 28	8.5
Cyclosulfamuron (环丙嘧磺隆)	422.0	261.1^*^, 218.1	102	9, 25	7.9
Chromafenozide (环虫酰肼)	395.1	175.0^*^, 339.0	90	2, 8	7.8
Hexazinone (环嗪酮)	253.1	171.0^*^, 71.2	96	13, 37	2.3
Pyriftalid (环酯草醚)	319.0	139.0^*^, 179.0	140	35, 35	6.9
Phorate (甲拌磷)	261.1	199.0^*^, 75.0	70	5, 10	9.3
Phorate sulfone (甲拌磷砜)	293.0	171.0^*^, 143.0	84	6, 10	6.1
Phorate sulfoxide (甲拌磷亚砜)	277.0	199.0^*^, 143.0	80	5, 15	5.9
Metsulfuron-methyl (甲磺隆)	382.0	167.1^*^, 199.0	90	15, 20	5.0
Mesosulfuron-methyl (甲基二磺隆)	504.1	182.1^*^, 139.1	125	25, 52	6.1
Phosfolan-methyl (甲基硫环磷)	228.0	168.0^*^, 109.0	90	14, 30	3.6
Pirimiphos-methyl (甲基嘧啶磷)	306.1	164.0^*^, 108.1	134	30, 33	9.2
Methiocarb (甲硫威)	226.2	169.0^*^, 121.0	80	5, 10	7.1
Methiocarb sulfone (甲硫威砜)	258.0	122.0^*^, 201.0	80	12, 6	4.1
Methiocarb sulfoxide (甲硫威亚砜)	242.1	185.0^*^, 122.0	75	14, 34	3.8
Carbaryl (甲萘威)	202.0	145.0^*^, 127.0	80	10, 30	5.4
Metalaxyl (甲霜灵)	280.1	220.0^*^, 192.0	96	9, 15	6.3
Methoxyfenozide (甲氧虫酰肼)	369.2	313.1^*^, 149.1	100	5, 9	12.5
Fenbuconazole (腈苯唑)	337.1	125.0^*^, 70.0	132	17, 33	8.3
Monocrotophos (久效磷)	224.1	127.0^*^, 193.0	100	20, 5	3.6
Carbofuran (克百威)	222.0	165.1^*^, 123.0	80	7, 17	5.2
Quizalofop-ethyl (喹禾灵)	373.1	255.1^*^, 271.2	130	36, 24	9.7
Dimethoate (乐果)	229.9	198.9^*^, 124.9	82	5, 17	4.1
Linuron (利谷隆)	249.0	182.0^*^, 160.0	96	6, 50	7.1
Cadusafos (硫线磷)	271.0	131.0^*^, 159.0	80	20, 10	9.3
Halosulfuron-methyl (氯吡嘧磺隆)	435.1	181.8^*^, 138.8	121	10, 46	7.5
Chlorimuron-ethyl (氯嘧磺隆)	415.0	186.0^*^, 213.0	120	10, 10	7.4
Malaoxon (马拉氧磷)	315.0	127.0^*^, 99.0	100	5, 20	5.2
Prochloraz (咪鲜胺)	376.1	308.0^*^, 266.0	80	10, 10	9.1
Fenamidone (咪唑菌酮)	312.0	236.0^*^, 92.0	100	10, 20	7.2
Dimoxystrobin (醚菌胺)	327.1	205.0^*^, 116.0	110	15, 25	8.6
Kresoxim-methyl (醚菌酯)	314.1	267.0^*^, 206.0	80	5, 5	8.7
Orthosulfamuron (嘧苯胺磺隆)	425.1	199.0^*^, 227.0	90	10, 14	6.3
Cyprodinil (嘧菌环胺)	226.1	108.1^*^, 93.1	152	25, 37	8.7
Azoxystrobin (嘧菌酯)	404.1	372.0^*^, 344.0	102	9, 25	7.0
Ethoprophos (灭线磷)	243.0	130.9^*^, 173.0	98	17, 9	8.1
Mepronil (灭锈胺)	270.1	228.1^*^, 119.0	108	9, 21	7.6
Chlorbenzuron (灭幼脲)	309.0	156.0^*^, 139.0	90	11, 30	8.7
Dimepiperate (哌草丹)	264.1	146.0^*^, 119.1	55	2, 18	9.5
Prometryn (扑草净)	242.1	200.0^*^, 158.0	136	17, 21	7.8
Clothianidin (噻虫胺)	250.2	132.0^*^, 169.1	80	15, 10	3.9
Thiacloprid (噻虫啉)	253.1	126.1^*^, 186.1	110	17, 10	4.5
Thiamethoxam (噻虫嗪)	292.1	211.0^*^, 181.0	80	5, 20	3.5
Thifensulfuron-methyl (噻吩磺隆)	388.0	167.1^*^, 205.0	115	12, 25	4.9
Buprofezin (噻嗪酮)	306.1	201.0^*^, 116.0	120	10, 15	9.8
Fosthiazate (噻唑磷)	284.1	228.1^*^, 104.0	86	5, 21	5.8
Tricyclazole (三环唑)	190.0	163.0^*^, 136.0	122	25, 29	4.5
Triazophos (三唑磷)	314.0	162.0^*^, 119.0	120	13, 41	7.8
Mandipropamid (双炔酰菌胺)	412.1	125.0^*^, 328.0	100	40, 10	7.3
Terbuthylazine (特丁津)	230.1	174.1^*^, 104.0	120	15, 20	7.2
Terbufos sulfone (特丁硫磷砜)	321.0	171.0^*^, 97.0	120	10, 35	6.9
Terbufos sulfoxide (特丁硫磷亚砜)	305.1	186.9^*^, 96.9	50	10, 50	6.9
Carboxin (萎锈灵)	236.0	143.0^*^, 87.1	92	13, 21	5.5
Trifloxystrobin (肟菌酯)	409.1	206.0^*^, 186.0	90	9, 13	9.5
Penoxsulam (五氟磺草胺)	484.1	195.1^*^, 164.1	149	25, 37	5.6
Penconazole (戊菌唑)	284.0	158.9^*^, 70.2	118	25, 13	8.7
Simetryn (西草净)	214.1	124.0^*^, 68.0	120	16, 40	6.0
Clethodim (烯草酮)	360.0	268.0^*^, 164.0	120	10, 20	9.6
Enestroburin (烯肟菌酯)	400.1	137.0^*^, 178.0	90	34, 14	9.8
Dimethomorph (烯酰吗啉)	388.0	301.0^*^, 165.0	120	20, 25	7.5
Amidosulfuron (酰嘧磺隆)	370.0	261.1^*^, 218.1	90	10, 24	5.5
Phoxim (辛硫磷)	299.1	129.0^*^, 77.0	80	10, 20	9.0
Diethofencarb (乙霉威)	268.0	226.0^*^, 152.0	80	5, 20	7.0
Ethoxysulfuron (乙氧磺隆)	399.1	261.1^*^, 218.1	112	9, 21	7.6
Isoproturon (异丙隆)	207.0	72.0^*^, 165.0	120	15, 15	6.3
Iprobenfos (异稻瘟净)	289.1	205.0^*^, 91.1	76	5, 17	8.5
Imazalil (抑霉唑)	297.0	159.0^*^, 201.0	126	21, 14	6.2
Coumaphos (蝇毒磷)	363.0	227.0^*^, 307.0	120	20, 15	8.9
Ametryn (莠灭净)	228.0	186.0^*^, 96.0	122	17, 30	6.9
Rotenone (鱼藤酮)	395.1	213.0^*^, 192.0	130	20, 25	8.4
Piperonyl butoxide (增效醚)	356.2	177.1^*^, 119.0	110	8, 37	9.9
Fenobucarb (仲丁威)	208.1	152.1^*^, 95.1	70	2, 9	7.0
Carfentrazone-ethyl (唑草酮)	412.0	346.0^*^, 366.0	120	20, 15	8.6

* Quantitative ion.

### 1.4 数据库的建立

本实验将118种农药的标准溶液按上述仪器分析条件进样,获得该条件下的母离子、碎裂电压、子离子、碰撞能量、保留时间,利用MassHunter软件建立118种农药数据库。

## 2 结果与讨论

### 2.1 提取方法的选择

为提高前处理工作效率,实验采用QuEChERS 法进行提取、净化。尿液基质主要成分为水分,除此之外还含有蛋白质、葡萄糖、尿酸、尿素以及无机盐等内源性物质^[21]^,这些内源性物质一方面会对痕量农药残留目标物的分析造成干扰,另一方面也会对色谱柱及质谱造成损害,需要有效的提取、净化方法去除杂质干扰。目前,农药残留检测前处理方法常用的提取试剂主要有乙腈、乙酸乙酯、丙酮等。其中乙酸乙酯与水较易分离,但对于强极性农药,其无法从含水基质中萃取完全。丙酮虽然可以从样品中很好地提取出残留农药,但同时也会提取出大量内源性干扰物质,另外其水溶性过强,难与基质中的水分分开,增加了分离难度。乙腈具有蛋白质沉淀作用,对尿液中的脂质溶解度低,利于在提取多组分农药的同时减少内源性基质干扰,另外其极性较大,穿透能力强,能够提取的农药范围宽、种类多,因此选用乙腈作为提取试剂。

尿液的pH值对于农药的提取效果有一定影响,文献报道有机磷农药、氨基甲酸酯类农药、苯氧乙酸类除草剂等在碱性条件下容易发生水解^[22,23]^。实验分别考察了3.0、5.0、7.0、9.0 4种pH条件下118种农药的提取效果,其中甲硫威砜、甲硫威亚砜在pH 9.0的条件下回收率低于20%。在尿液样品中加入盐酸使尿液保持酸性有利于防止部分农药因尿液碱化而分解,抑制苯氧羧酸类除草剂等农药电离,提高溶剂提取率。总体来看,pH 5.0条件下118种农药的提取回收率优于pH 3.0。综合考虑,实验最终采用加入1 mol/L盐酸调节尿样pH至5.0,再加入乙腈提取。

QuEChERS样品前处理方法常用的盐析剂包括NaCl、无水MgSO_4_等。其中加入NaCl可以调节溶液的极性从而影响液液分配,有利于极性农药的萃取。无水MgSO_4_与水发生水合作用释放热量,可以使萃取溶液的温度达到40~45 ℃,有利于农药的萃取。通过对比,单独使用5 g NaCl时,有机相与水相能够分离,但部分农药提取回收率低于80%;单独使用5 g无水MgSO_4_时,水分不能完全除去且与有机相不能有效分离。而在联合使用5 g无水MgSO_4_及1 g NaCl时,更加有利于有机相与水相的分离和农药的提取,118种农药的回收率均高于80%。综合考虑,实验选用5 g无水MgSO_4_和1 g NaCl作为除水盐析剂。

### 2.2 净化方法的选择

目前常见的QuEChERS净化吸附剂包括C_18_、PSA、石墨化炭黑(GCB)及无水MgSO_4_,其中C_18_对脂类、甾醇等非极性化合物有较强的吸附作用,PSA可通过氨基的弱离子交换作用和极性基质成分形成氢键,从而吸附和消除糖类、色素、有机酸及金属离子等杂质,GCB主要用于去除甾醇和弱极性色素,但对非极性和具有平面结构的农药也有一定的吸附,无水MgSO_4_一般作为除水剂^[24⇓⇓-27]^。实验发现PSA、C_18_、MgSO_4_对所选取的118种农药化合物没有明显的吸附作用,在尿样提取液中加入C_18_、PSA和无水MgSO_4_,可获得良好的净化效果,而GCB对于多菌灵等非极性和具有平面结构的农药吸附明显。取6 mL提取液,对比150 mg C_18_+150 mg PSA+900 mg无水MgSO_4_、 300 mg C_18_+300 mg PSA+900 mg无水MgSO_4_、 600 mg C_18_+600 mg PSA+900 mg无水MgSO_4_ 3种吸附剂组合的净化效果,结果见图1。结果表明,在使用300 mg C_18_+300 mg PSA+900 mg无水MgSO_4_的条件下,基质干扰可得到有效降低,且118种农药的基质效应均低于20%,满足实验要求;而使用600 mg C_18_+600 mg PSA+900 mg无水MgSO_4_的吸附剂组合时,26%的农药受到吸附影响,回收率有一定降低。最终净化方法选用300 mg C_18_+300 mg PSA+900 mg无水MgSO_4_作为吸附剂。

**图1:**
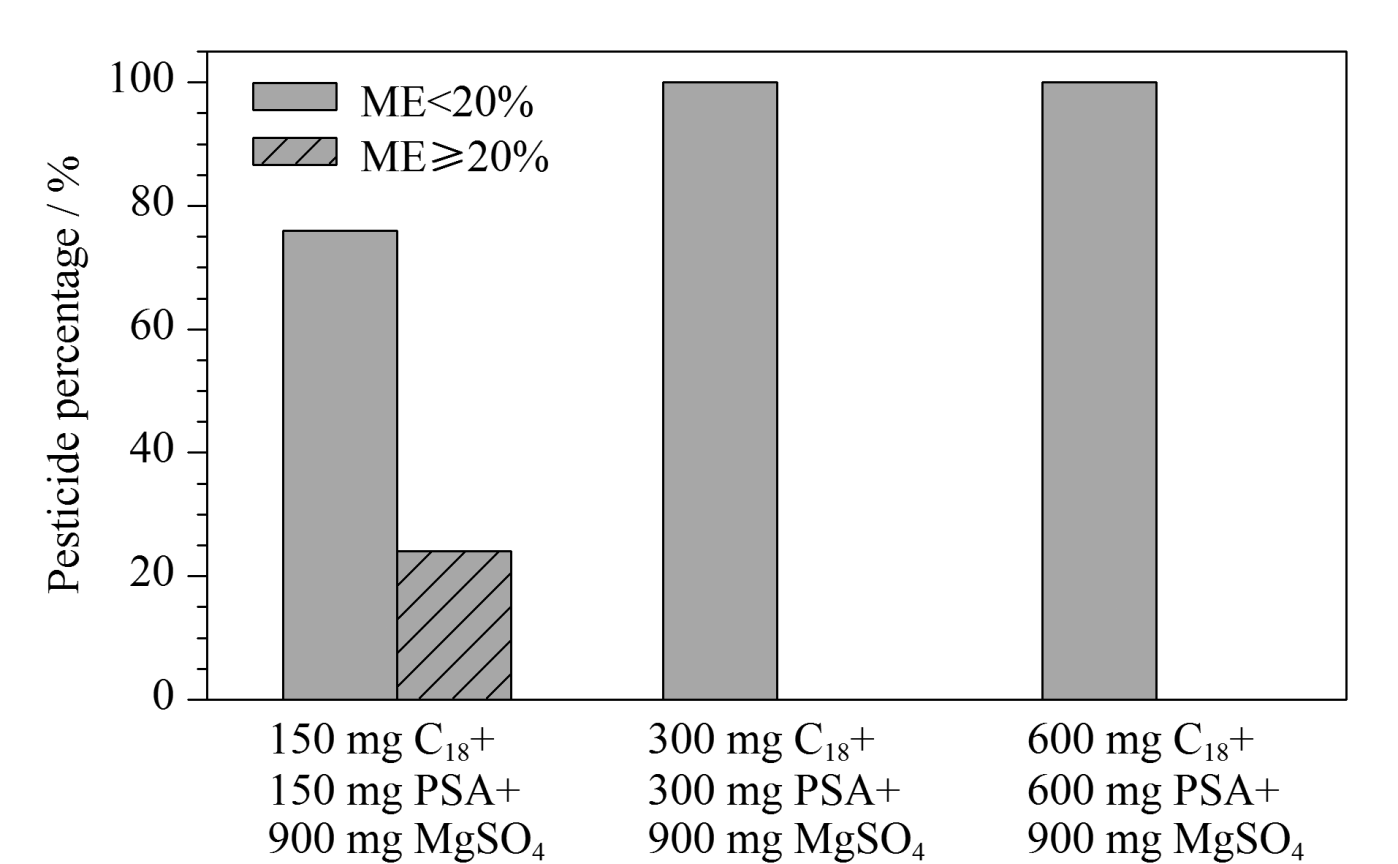
经不同吸附剂组合净化后两个基质效应水平的农药比例

### 2.3 色谱-质谱条件优化

本实验选用ZORBAX Eclipse Plus C_18_作为分离柱,有机相为0.01%甲酸甲醇溶液(含2 mmol/L甲酸铵),水相为0.01%甲酸水溶液(含2 mmol/L甲酸铵)。在流动相中加入0.01%甲酸能够显著提高各农药的响应值,加入2 mmol/L甲酸铵后能够减少色谱峰拖尾,获得更好的峰形。通过优化,采用1.3节中梯度洗脱条件,118种农药实现了良好的分离,各化合物的保留时间见表1。

分别采用正离子扫描模式(ESI^+^)和负离子扫描模式(ESI^-^)对118种农药标准溶液进行母离子扫描。除氟虫腈、氟虫腈砜在负离子扫描模式下得到准分子离子峰外,其余农药采用正离子扫描模式均可产生稳定的[M+H]^+^准分子离子峰。进一步对118种农药的子离子、碎裂电压、碰撞能量等参数进行优化,优化后的质谱分析参数详见表1。利用MassHunter软件建立118种农药数据库,调取数据库信息建立DMRM方法。

### 2.4 基质效应的评价

基质效应是指样品中除了目标分析物以外的其他成分对待测物测定值的影响。使用ESI作为电离源时,容易出现离子化抑制现象,常常对分析物的电离有显著的干扰,并影响分析结果的准确性。实验分别用溶剂和空白基质溶液配制1、2.5、5、10、25、50 μg/L混合标准溶液,以峰面积对相应的质量浓度做标准曲线,然后比较这两条标准曲线斜率的差异,从而判断基质效应的强弱。计算公式:基质效应=(基质匹配标准曲线的斜率/溶剂标准曲线的斜率-1)×100%。结果如表2所示,118种农药的基质效应均为基质抑制效应,且基质效应均小于20%,采用溶剂标准溶液即可进行定量。

**表2:** 尿液中118种农药的决定系数(*r*^2^)、回收率、相对标准偏差(RSD)、检出限、定量限及基质效应(*n*=6)

Compound	r^2^	0.50 μg/L			1.00 μg/L			5.00 μg/L		LOD/ (μg/L)	LOQ/ (μg/L)	ME/ %
		Recovery/%	RSD/%		Recovery/%	RSD/%		Recovery/%	RSD/%			
Fenpyrazamine	0.9993	87.0	6.9		89.8	6.5		94.6	5.5	0.10	0.50	-4.5
Pyraclostrobin	0.9999	92.3	8.8		96.6	7.2		99.7	6.7	0.10	0.50	-8.5
Fenthion	0.9999	73.3	6.8		77.2	6.6		81.1	5.4	0.10	0.50	-8.3
Fenthion sulfoxide	0.9997	80.2	6.6		84.2	6.7		92.3	5.5	0.10	0.50	-8.8
Tribenuron-methyl	0.9999	82.0	8.9		85.4	8.7		96.7	6.1	0.10	0.50	-5.5
Metrafenone	0.9999	75.0	7.3		80.0	6.7		85.1	5.1	0.10	0.50	-8.7
Difenoconazole	0.9992	75.9	7.9		82.8	6.4		86.0	5.4	0.10	0.50	-7.6
Mefenacet	0.9994	72.6	8.5		78.8	8.3		83.9	6.8	0.10	0.50	-11.8
Benalaxyl	0.9994	79.0	5.3		82.7	5.0		85.9	3.6	0.10	0.50	-5.2
Zoxamide	0.9994	88.5	9.3		90.1	8.6		101	8.1	0.10	0.50	-3.4
Fenamiphos	0.9991	78.9	6.8		84.3	6.6		88.7	6.1	0.10	0.50	-7.0
Imidacloprid	0.9991	90.4	4.1		94.1	4.0		100	3.5	0.10	0.50	-3.2
Diflufenican	0.9996	72.0	7.4		77.7	6.4		84.0	4.9	0.10	0.50	-11.5
Penthiopyrad	0.9995	86.7	4.4		88.1	4.2		95.4	3.5	0.10	0.50	-4.8
Metazachlor	0.9991	81.7	6.8		83.6	6.1		90.0	5.2	0.10	0.50	-8.5
Isopyrazam	0.9998	84.9	8.2		90.8	7.4		96.4	6.9	0.10	0.50	-7.9
Prosulfocarb	0.9995	82.2	9.0		88.9	7.9		93.1	7.3	0.10	0.50	-8.1
Bensulfuron-methyl	0.9999	72.8	5.2		77.0	5.1		83.2	4.3	0.10	0.50	-6.5
Pretilachlor	0.9997	84.6	8.6		89.2	8.2		96.5	7.8	0.10	0.50	-8.2
Propyrisulfuron	1.0000	81.8	7.6		88.6	7.5		92.8	6.6	0.10	0.50	-8.1
Propoxur	0.9993	89.8	4.3		93.3	3.7		97.0	3.7	0.10	0.50	-4.1
Tebufenozide	0.9993	89.3	5.5		93.2	5.4		95.9	3.8	0.10	0.50	-12.4
Pyridaphenthion	0.9998	87.1	6.2		88.6	5.2		96.3	5.7	0.10	0.50	-11.0
Isoprothiolane	0.9992	77.6	5.1		82.6	4.5		88.2	3.1	0.10	0.50	-7.7
Edifenphos	0.9998	85.8	6.0		91.3	5.8		96.2	4.4	0.10	0.50	-9.6
Tebuthiuron	1.0000	80.8	5.4		83.9	3.9		86.2	2.8	0.10	0.50	-8.0
Acetamiprid	0.9995	86.6	6.5		89.9	4.7		95.0	4.4	0.10	0.50	-3.9
Picoxystrobin	0.9992	80.7	4.6		84.0	3.6		87.7	3.0	0.10	0.50	-11.8
Chlorfenvinphos	0.9995	80.8	8.8		87.3	7.6		96.2	6.3	0.10	0.50	-8.5
Carbendazim	0.9993	83.2	7.9		87.2	7.5		91.5	7.0	0.10	0.50	-4.1
Metamifop	0.9993	95.2	6.3		97.7	6.1		104	5.2	0.10	0.50	-3.1
Diazinon	0.9994	87.9	6.9		91.0	6.2		93.6	5.0	0.10	0.50	-5.9
Fensulfothion	0.9997	70.7	6.0		74.1	5.9		80.7	5.1	0.10	0.50	-12.8
Dinotefuran	0.9999	84.9	8.5		88.3	7.8		95.7	5.6	0.10	0.50	-3.6
Furathiocarb	0.9991	82.8	8.5		89.1	7.4		93.8	6.8	0.10	0.50	-5.3
Flucetosulfuron	0.9994	76.8	6.0		80.2	5.6		85.5	5.1	0.10	0.50	-7.5
Fluopicolide	0.9993	83.7	5.4		86.1	5.0		93.2	3.5	0.10	0.50	-7.2
Fluopyram	0.9999	89.5	8.4		91.7	7.7		98.1	5.7	0.10	0.50	-8.2
Fipronil	0.9995	84.2	9.2		89.4	8.2		93.0	7.6	0.10	0.50	-4.9
Fipronil sulfone	0.9993	73.3	9.0		75.9	8.5		89.7	8.2	0.10	0.50	-11.3
Flusilazole	0.9992	83.2	8.0		86.6	7.9		91.9	7.7	0.10	0.50	-8.5
Triflumizole	0.9997	88.4	4.8		90.7	4.6		94.2	3.3	0.10	0.50	-10.8
Flufenacet	0.9991	74.5	6.3		76.9	5.6		83.5	5.3	0.10	0.50	-11.2
Flutolanil	0.9996	84.0	8.8		87.8	8.3		94.3	7.5	0.10	0.50	-8.1
Fluxapyroxad	0.9994	73.3	8.2		78.0	6.3		83.4	5.5	0.10	0.50	-10.0
Silthiofam	0.9997	77.5	6.9		81.1	5.5		84.0	4.9	0.10	0.50	-9.0
Cyclosulfamuron	0.9995	73.8	7.2		76.5	6.4		81.7	5.7	0.10	0.50	-11.7
Chromafenozide	0.9997	83.1	8.8		86.6	7.4		90.2	7.0	0.10	0.50	-3.9
Hexazinone	0.9998	76.6	7.8		81.9	7.4		87.0	6.3	0.10	0.50	-12.4
Pyriftalid	0.9999	75.4	8.9		79.7	8.2		86.5	7.4	0.10	0.50	-11.6
Phorate	0.9991	74.8	8.0		79.4	8.5		83.5	5.6	0.10	0.50	-7.1
Phorate sulfone	0.9991	88.1	8.3		93.0	7.7		99.5	6.7	0.10	0.50	-7.9
Phorate sulfoxide	0.9995	76.0	7.0		80.8	6.8		88.1	5.6	0.10	0.50	-9.4
Metsulfuron-methyl	0.9996	85.6	6.0		88.3	5.7		95.0	3.7	0.10	0.50	-11.0
Mesosulfuron-methyl	0.9993	83.7	6.6		86.7	5.8		97.1	4.7	0.10	0.50	-7.3
Phosfolan-methyl	0.9999	74.2	8.1		78.5	7.7		83.7	7.0	0.10	0.50	-12.0
Pirimiphos-methyl	0.9992	72.8	6.7		78.8	6.5		82.8	5.5	0.10	0.50	-7.9
Methiocarb	0.9994	79.7	7.0		83.6	6.9		86.9	6.8	0.10	0.50	-5.0
Methiocarb sulfone	0.9999	76.2	6.8		79.5	6.6		88.2	5.6	0.10	0.50	-7.5
Methiocarb sulfoxide	0.9997	71.9	7.9		74.5	7.1		86.5	5.9	0.10	0.50	-12.4
Carbaryl	0.9993	83.1	6.0		87.9	5.2		94.1	5.0	0.10	0.50	-7.3
Metalaxyl	0.9998	89.3	8.2		91.3	7.3		98.2	6.8	0.10	0.50	-6.2
Methoxyfenozide	0.9996	70.2	8.1		74.2	7.4		80.6	6.5	0.10	0.50	-8.6
Fenbuconazole	0.9996	81.8	8.1		83.6	6.9		94.0	5.9	0.10	0.50	-6.4
Monocrotophos	0.9993	73.5	5.4		76.9	5.4		80.8	4.3	0.10	0.50	-4.5
Carbofuran	0.9991	89.7	8.3		95.0	7.2		97.3	6.1	0.10	0.50	-3.3
Quizalofop-ethyl	1.0000	78.3	7.8		82.1	6.7		87.7	5.0	0.10	0.50	-8.8
Dimethoate	0.9991	82.9	5.2		86.6	5.6		92.8	4.6	0.10	0.50	-8.3
Linuron	0.9991	77.7	6.2		81.2	6.7		84.3	4.7	0.10	0.50	-12.6
Cadusafos	0.9992	83.2	5.7		87.9	5.3		93.5	3.7	0.10	0.50	-6.9
Halosulfuron-methyl	0.9997	71.3	9.3		77.6	9.2		83.1	7.2	0.10	0.50	-8.4
Chlorimuron-ethyl	1.0000	76.2	7.5		82.1	6.9		85.3	4.4	0.10	0.50	-8.9
Malaoxon	0.9996	74.9	5.2		80.3	4.9		83.0	3.5	0.10	0.50	-12.7
Prochloraz	0.9993	84.7	8.7		89.0	8.1		94.5	6.1	0.10	0.50	-6.9
Fenamidone	0.9994	84.5	7.5		87.6	6.9		97.2	5.7	0.10	0.50	-8.6
Dimoxystrobin	0.9993	75.5	8.7		81.1	8.2		86.4	7.1	0.10	0.50	-6.7
Kresoxim-methyl	0.9993	91.4	8.5		94.5	8.1		101	7.1	0.10	0.50	-6.8
Orthosulfamuron	0.9991	87.2	5.7		90.0	5.2		94.6	3.8	0.10	0.50	-7.0
Cyprodinil	1.0000	71.5	4.5		75.2	4.7		78.6	4.2	0.10	0.50	-12.3
Azoxystrobin	0.9993	76.2	7.3		78.8	6.0		83.4	5.1	0.10	0.50	-12.4
Ethoprophos	0.9994	81.9	6.7		84.7	5.1		87.8	3.5	0.10	0.50	-10.2
Mepronil	0.9998	80.3	7.6		82.6	6.8		91.6	6.4	0.10	0.50	-4.8
Chlorbenzuron	0.9991	87.5	7.0		93.0	6.5		96.0	6.1	0.10	0.50	-6.1
Dimepiperate	0.9997	72.2	5.3		74.8	5.5		77.7	4.2	0.10	0.50	-7.6
Prometryn	1.0000	84.2	5.4		89.0	4.2		93.6	3.6	0.10	0.50	-3.9
Clothianidin	0.9992	89.1	8.1		95.4	7.2		101	4.9	0.10	0.50	-3.2
Thiacloprid	0.9991	70.7	7.3		72.8	6.9		78.7	5.2	0.10	0.50	-12.3
Thiamethoxam	0.9999	80.5	4.1		87.5	4.3		89.9	3.8	0.10	0.50	-12.8
Thifensulfuron-methyl	0.9994	83.7	8.9		86.7	8.5		93.6	7.2	0.10	0.50	-12.2
Buprofezin	0.9992	74.2	6.1		77.0	5.4		80.7	5.2	0.10	0.50	-8.1
Fosthiazate	0.9997	88.5	7.6		90.5	7.3		101	6.0	0.10	0.50	-3.1
Tricyclazole	0.9992	85.5	8.9		92.3	7.7		98.7	6.0	0.10	0.50	-5.6
Triazophos	0.9991	76.6	6.9		80.2	6.6		83.3	5.6	0.10	0.50	-7.7
Mandipropamid	0.9998	79.0	7.1		81.0	6.8		85.5	4.9	0.10	0.50	-4.9
Terbuthylazine	0.9996	86.3	7.2		88.8	6.0		93.2	5.5	0.10	0.50	-7.5
Terbufos sulfone	0.9998	79.3	7.3		82.1	6.3		91.6	5.3	0.10	0.50	-4.1
Terbufos sulfoxide	0.9992	79.1	8.6		84.4	7.5		90.7	6.9	0.10	0.50	-9.4
Carboxin	0.9993	77.9	6.3		81.0	4.4		85.0	3.8	0.10	0.50	-12.1
Trifloxystrobin	0.9995	80.8	7.4		85.0	6.8		92.3	6.2	0.10	0.50	-9.9
Penoxsulam	0.9998	81.0	8.4		86.1	8.2		90.0	7.3	0.10	0.50	-12.0
Penconazole	0.9997	76.4	7.1		82.1	5.9		84.2	5.6	0.10	0.50	-5.8
Simetryn	0.9993	83.5	6.7		88.8	6.6		97.4	5.3	0.10	0.50	-8.6
Clethodim	0.9993	87.5	8.4		92.7	7.8		95.3	6.3	0.10	0.50	-6.3
Enestroburin	0.9997	78.2	7.8		82.8	7.2		84.8	5.7	0.10	0.50	-12.4
Dimethomorph	1.0000	82.9	5.1		86.8	5.9		93.5	4.2	0.10	0.50	-3.0
Amidosulfuron	0.9991	86.4	5.2		89.3	5.1		93.3	3.4	0.10	0.50	-9.4
Phoxim	0.9995	80.8	6.4		84.4	5.7		88.7	4.3	0.10	0.50	-6.1
Diethofencarb	0.9998	88.0	4.1		92.1	4.5		101	3.3	0.10	0.50	-3.6
Ethoxysulfuron	0.9992	73.4	7.9		78.9	7.0		82.1	5.6	0.10	0.50	-5.5
Isoproturon	0.9995	86.7	9.0		88.6	8.8		95.2	7.2	0.10	0.50	-3.7
Iprobenfos	0.9994	80.9	7.3		86.0	6.8		91.7	5.8	0.10	0.50	-11.7
Imazalil	0.9994	79.3	6.6		81.0	5.3		88.7	4.7	0.10	0.50	-12.3
Coumaphos	0.9994	80.1	7.3		82.8	6.8		89.6	5.5	0.10	0.50	-11.1
Ametryn	0.9993	83.7	7.9		87.2	6.0		91.5	4.8	0.10	0.50	-7.5
Rotenone	0.9997	80.3	6.1		83.7	5.1		90.3	4.6	0.10	0.50	-10.2
Piperonyl butoxide	0.9995	90.5	8.1		93.5	7.0		98.1	5.4	0.10	0.50	-4.9
Fenobucarb	0.9994	82.0	6.2		85.4	5.9		90.2	4.2	0.10	0.50	-11.7
Carfentrazone-ethyl	0.9992	87.7	6.5		89.9	6.1		96.3	5.9	0.10	0.50	-5.2

### 2.5 方法的线性关系、定量限、回收率和精密度

配制1、2.5、5、10、25、50 μg/L的系列标准溶液,在已建立的分析条件下进行测定,以峰面积(*y*)对质量浓度(*x*)做标准曲线,118种农药的决定系数均大于0.999(见表2),表明线性关系良好。以空白尿液样品加标,按本方法前处理后分析检测,当样品中农药含量大于0.10 μg/L时,仪器信噪比均大于3,当样品中农药含量大于0.50 μg/L时,仪器信噪比均大于10,因此确定检出限为0.10 μg/L,定量限为0.50 μg/L。

向空白尿液样品中添加118种农药的混合标准溶液,添加水平分别为0.50、1.00、5.00 μg/L,然后按照1.2节和1.3节方法进行提取、净化和检测。每个水平重复6次试验,118种农药的平均回收率为70.2%~104%,相对标准偏差为2.8%~9.3%(见表2)。

### 2.6 实际样品的检测

应用本文所建立的方法对10份尿液样品进行检测,将检出的色谱峰保留时间、离子丰度比分别与标准品色谱峰的保留时间、离子丰度比相比较,经确证,共检出6种农药,保留时间偏差均在±0.1 min之内,离子丰度比偏差均在±20%内,检测结果见表3,代表性MRM图谱如图2所示。

**表3:** 10份尿液样品的检测结果

Sample No.	Pesticide residues/(μg/L)					
	Thiamethoxam	Clothianidin	Acetamiprid	Dinotefuran	Isoproturon	Dimethomorph
1	<LOQ	<LOQ	-	0.61	-	-
2	1.30	1.13	-	-	-	-
3	1.28	1.15	-	<LOQ	0.53	0.72
4	0.93	0.52	-	-	-	-
5	1.31	3.65	-	-	-	-
6	<LOQ	0.62	0.58	-	-	-
7	0.83	3.36	-	-	-	-
8	<LOQ	<LOQ	-	-	-	-
9	<LOQ	0.85	-	-	-	-
10	-	<LOQ	-	-	-	-

-: not detected.

**图2:**
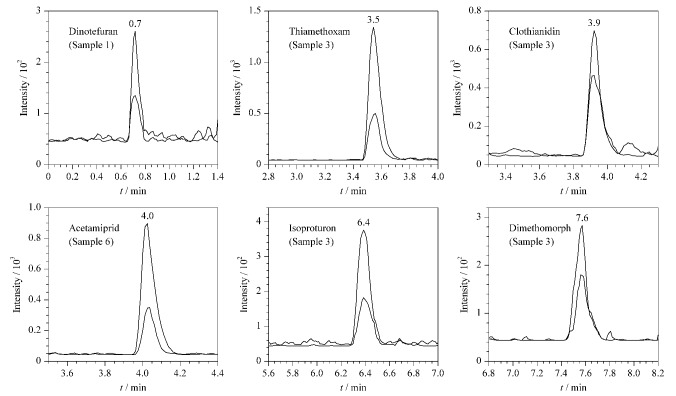
尿液样品中6种农药的代表性MRM色谱图

由于样品来自同一区域,10份尿样检出农药比较集中,以新烟碱类农药为主,其中10份尿样均检出农药噻虫胺,2份尿样的农药含量小于定量限,其余尿样的农药含量分布在0.52~3.65 μg/L。9份尿样检出农药噻虫嗪,其中4份尿样含量小于定量限,其余尿样的农药含量分布在0.83~1.31 μg/L。2份尿样检出农药呋虫胺,其中1份尿样的农药含量小于定量限,1份尿样的农药含量为0.61 μg/L。1份尿样检出农药啶虫脒,含量为0.58 μg/L。另外尿样3共检出5种农药,除检出新烟碱类农药外,还检测到异丙隆和烯酰吗啉,分别为0.53 μg/L和0.72 μg/L。噻虫嗪、噻虫胺是高效低毒杀虫剂,噻虫嗪也可以代谢转化为噻虫胺。尿液中检出噻虫嗪、噻虫胺频次较高与当前食用农产品生长过程中该类农药使用情况关联性较高,如近些年市场监管部门抽检公布的食用农产品抽检不合格信息中^[28⇓-30]^,香蕉、辣椒、姜、豇豆等果蔬中噻虫嗪、噻虫胺不合格情况时有发生,有的甚至超标严重,需引起重视。

## 3 结论

本实验建立了检测尿液中118种农药残留的超高效液相色谱-串联质谱方法。样品中农药采用乙腈提取,以C_18_、PSA、无水MgSO_4_为净化吸附剂经QuEChERS法净化,采用UHPLC-MS/MS检测,外标法定量。结果表明,该方法可以同时对尿液中118种农药进行快速筛查及准确测定。该方法快速、准确,分析通量高,可以为人尿液中多农药残留的快速筛查和准确定性定量提供方法依据。
